# Metagenomic analysis of mosquitoes from Kangerlussuaq, Greenland reveals a unique virome

**DOI:** 10.1038/s41598-025-01086-z

**Published:** 2025-05-17

**Authors:** Mirjam Schilling, Madhujot Jagdev, Huw Thomas, Nicholas Johnson

**Affiliations:** 1 Virology Department, Animal and Plant Health Agency, Woodham Lane, Addlestone, KT15 3 NB Surrey UK; 2 Greenland White-fronted Goose Study group/VEO Project, Bristol, UK

**Keywords:** *Aedes impiger*, *Aedes nigripes*, Arctic, Climate, Vector, Bioinformatics, Microbial genetics, Virology, Genomics, Sequencing, Ecology, Climate sciences

## Abstract

Climate change is dramatically affecting vector ecology in extreme environments such as the Arctic. However, little is known about the current status of viruses of arthropod vectors located in such northerly locations. As part of a field survey on the role of wildlife in international movement of zoonotic pathogens, we sampled mammalophilic mosquitoes near the settlement of Kangerlussuaq, Greenland in July 2022 and July 2023 to investigate their virome. The majority of mosquitoes were identified as either *Aedes impiger* or *Aedes nigripes*. Metagenomic analysis of RNA extracted from species pools detected a number of novel RNA viruses belonging to a range of different virus families, including Flaviviridae, Orthomyxoviridae, Bunyavirales, Totiviridae and Rhabdoviridae. However, the sequence identities when compared to previously published, were as low as 34% at the amino acid level. Furthermore, a comparison of virome diversity between *Aedes* species emphasises the uniqueness of both *Aedes impiger* and *Aedes nigripes* from this secluded ecosystem. It also highlights the need to better understand the viromes of potential pathogen vectors as the impacts of climate change are experienced in such northerly ecosystems.

**Supplementary Information:**

The online version contains supplementary material available at 10.1038/s41598-025-01086-z.

## Introduction

Global changes in climate are causing a shift in the distribution of vectors^1^ and increasing the likelihood of vector-borne disease outbreaks in regions that have not experienced such disease emergence^2,3^. Yet, very little is known about the vectorial capacity and virome of mosquitoes in less accessible areas such as the Arctic. In addition to a potential change in the localisation of Arctic insects in the long term, climate change already affects vectors in their current locations. With rapid environmental change, and warming at twice the global average^4^ the ecology of Arctic insects will be dramatically affected. Arctic insects have uniquely adapted to long, cold winters and short, cool, unpredictable summers^5^. Despite this, mosquito species have established in Arctic locations and are voracious feeders readily feeding on humans if present^6^. Due to the different ways a warming climate will affect the microclimate that they inhabit, the interplay of factors impacting Arctic mosquitoes can be complex. How climate change exactly impacts the vector ecology in the Arctic is therefore hard to predict. Nevertheless, we can be sure that all aspects of the Arctic insect life will be affected, including survival, development time, life cycle, host-seeking activity, interactions with other species, and range expansions^6^. This necessarily comes with implications for the entirety of northern ecosystems as well as the importance of Arctic mosquitoes as disease vectors. Additionally, a warming climate might also favour species introduction into the Arctic through an increase in tourism and travel, causing additional concerns around the implications of the diseases they carry for the local ecosystems and indigenous populations^7,8^. Newly arriving diseases could disproportionally impact Greenland’s wildlife, because their populations are likely immunologically naïve^9^.

Metagenomic analysis based on mass sequencing is an established methodology for determining the virome of arthropod species^10^. This approach has been used extensively for analysis of the viral composition of mosquito species associated with pathogen transmission, particularly those within the genus *Aedes*^11–14^. Understanding the diversity of the mosquito virome is a critical first step that can determine the relationship between known pathogens and the insect-only virus composition of mosquitoes that in turn could lead to novel strategies of control of mosquito-borne disease^15^. However, studies of the virome of indigenous mosquito species in extreme northernly locations have been limited^16,17^.

The aim of this study was to explore the virome of the local mosquito population in Greenland, applying a metagenomics approach. The few studies conducted in Greenland so far identified *Aedes nigripes* (Zetterstedt, 1838) and *Aedes impiger* (Walker, 1848) as the main native mosquito species^18^, but did not detect any arthropod-vectored viruses^19,20^. However, exploring the entirety of the mosquito virome, including both insect specific viruses as well as potential arboviruses will enable us to better understand the risks a change in location of Arctic mosquitoes might harbour, but even more importantly, take us one step closer to estimating the vector competence of so far understudied mosquito species for relevant virus families. Our study highlights the importance of exploring the virome in secluded locations such as the Arctic as climate change is already causing dramatic changes in species distribution that is accompanied by a shift in pathogen localisation.

## Materials and methods

### Mosquito collection

**Fig. 1:**
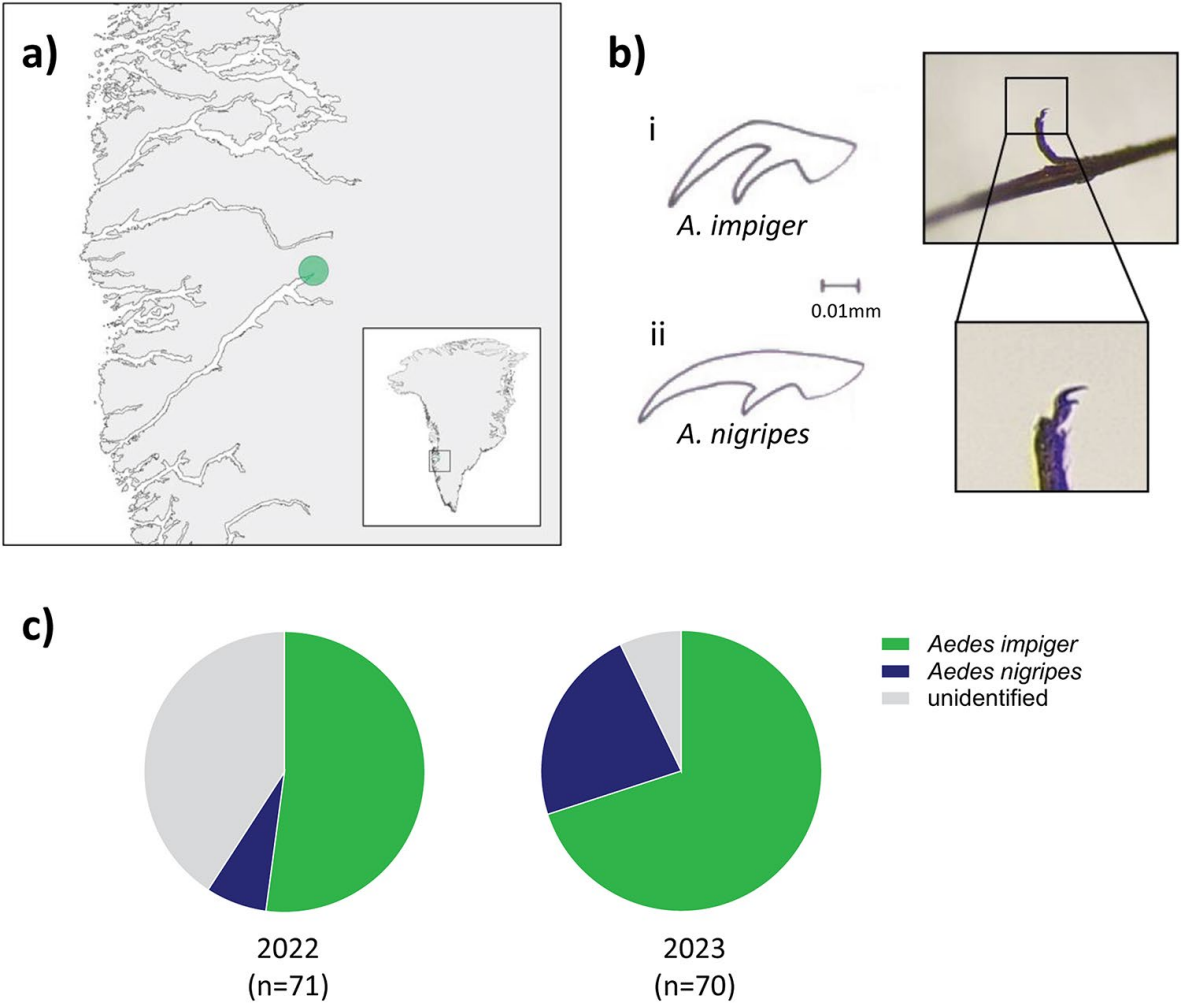
*Aedes impiger* was the predominant species collected in Kangerlussuaq, Greenland. **(a)** Map of Greenland depicting the sampling location at lake Sanningasoq, approximately 11.5 km northeast from Kangerlussuaq. The map was produced with ggplot2 (version 3.5.1) in R (version 4.4.1). **(b)** Species identification by morphology, Danks and Corbet, 1973. Image shows the tarsal claw of an *Aedes impiger* sampled in 2022. **(c)** Pie charts representing the number of mosquitoes identified for each year and species by DNA barcoding.

Mosquitoes were trapped at an open air camp at lake Sanningasoq, approximately 11.5 km northeast from Kangerlussuaq (67°01’N 50°41’W) in central-western Greenland (Fig. 1a), from 05.07. to 25.07.2022 (14 sampling days) and from 04.07. to 22.07.2023 (seven sampling days) using aspirators (Tubular suction aspirator, 7 mm intake, model D-601, Entomopraxis, Barcelona). They were killed by pipe smoke and kept at low temperature by immersing the collection tubes in a lake (estimated temperature between 3 ºC and 6 ºC). To prevent nucleic acid degradation, in 2023 a total of 70 mosquitoes were instantly homogenised individually in DNA/RNA Shield (Zymo) after trapping by shaking them in a 1.5 ml Eppendorf tube with a 5 mm stainless steel bead (Qiagen, Manchester, UK).

### Morphological identification of mosquitoes

Mosquito species identification by morphology was carried out under a microscope (Leica M165 C) following the guide by Danks and Corbet^18^. For *Aedes nigripes* the tarsal claw curves gradually, while for *Aedes (A) impiger* the tarsal claw curves abruptly (Fig. 1B).

### Molecular identification of mosquitoes

To confirm the morphological species identification and to identify specimens where the tarsal claws were missing, cytochrome c oxidase I (*cox*1) sequences were obtained.

In the pilot project i.e., mosquitoes trapped in 2022, two legs were used to extract DNA for species identification by DNA barcoding. Total DNA was extracted using DNeasy kits (Qiagen, Manchester, UK) according to the manufacturer’s instructions. Mosquitoes were then homogenised individually in 350 µl tissue culture medium using the Qiagen TissueLyser II with 5 mm stainless steel beads (both Qiagen, Manchester, UK) and centrifuged (10,000 rpm/10 min). Total RNA was extracted from 250 µl of the supernatant using RNeasy kits (Qiagen, Manchester, UK). The precipitated RNA was resuspended in 40 µl nuclease free water and pooled by species and date for next generation sequencing (NGS).

In 2023, to prevent nucleic acid degradation, mosquitoes were homogenised individually in DNA/RNA Shield (Zymo) immediately after trapping. DNA and RNA were then extracted separately using the AllPrep DNA/RNA Mini Kit (Qiagen, Manchester, UK). The precipitated DNA and RNA were resuspended in 40 µl nuclease free water. DNA was used for species identification by DNA barcoding and RNA was later pooled by species and sampling date for further analysis by NGS.

A 658 bp region located at the 5’ end of the *cox*1 gene was amplified by PCR with the primer pair (LCO1490 and HCO2198) published by Folmer et al.^21^. PCR products were visualised on a 1.5% agarose gel and samples of the correct band size were submitted for Sanger sequencing using primers LC01490 and HCO2198. Mosquito species were identified following a BLASTN search. Sequence identities were > 99% when compared with published Aedes impiger (Genbank: JN303080) and Aedes nigripes (Genbank: KR395472) sequences.

### Next generation sequencing

Extracted mosquito RNA was pooled based on species and date of trapping and subjected to next generation sequencing for metagenomic analysis (Supplementary Tables 1 and 2). Sequencing libraries were prepared using the Nextera XT kit (Illumina, Cambridge, UK) and analysed on a NextSeq sequencer (Illumina, Cambridge, UK) with 2 × 150 base paired-end reads.

### Data analysis

Reads were analysed using the Chan Zuckerberg Illumina pipeline^22^, a cloud-based, open-source bioinformatics platform: Reads were aligned against NCBI NT and NR databases using Minimap2 and Diamond, contigs were assembled with SPAdes, reads mapped against contigs using Bowtie2 and contigs aligned against nucleotide and protein databases with BLASTN and BLASTX. According to the Chan Zuckerberg pipeline (for all projects created prior April 19, 2023), the Host Filtering and Quality Control steps included initial host filtration using STAR, trimming of sequencing adapters using Trimmomatic, quality filtering using PriceSeq, identification of duplicate reads using czid-dedup, filtering out of low complexity sequences using LZW, filtering out remaining host sequences using Bowtie2, subsampling to 1 million fragments (reads/read-pairs) if > 1 M remain after step, and filtering out human sequences, regardless of host (using STAR, Bowtie2, and GSNAP). Details of the total reads per sample, the percentage that passed QC, duplicate compression ratio (DCR) and the number of reads that remained after host filtering can be found in Supplementary Table 3.

Hits that produced a minimum of one contig were investigated further. If multiple contigs were recovered from the same virus, the longest contig was investigated further.

The map depicting the trapping location was produced with ggplot2 (version 3.5.1) in R (version 4.4.1).

**Fig. 2:**
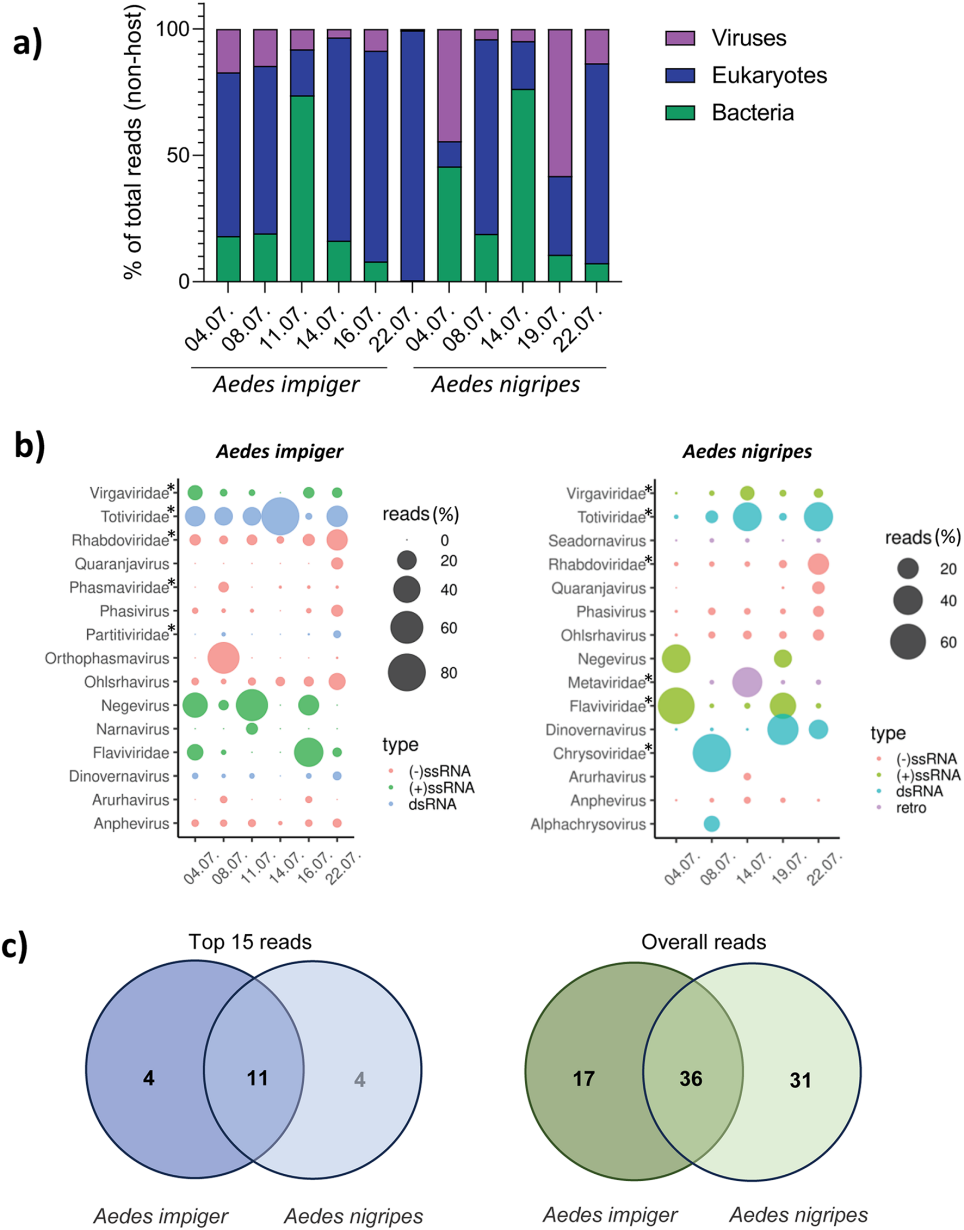
Distribution of non-host reads. **(a)** Distribution of non-host reads across the 2023 mosquito pools for each indicated sampling date and species. **(b)** Bubble plots representing the top15 viral hits (genus-level) of mosquito pools sampled in 2023 in percentage by species and date. The asterix marks non-genus specific reads in a virus family. **(c)** Overlap of viral hits (genus level) between the species across the top 15 reads only (left) or all viral reads (right).

The bubble blots in Fig. 2 were made with ggplot2 (version 3.5.1) in R (version 4.4.1).

**Fig. 3:**
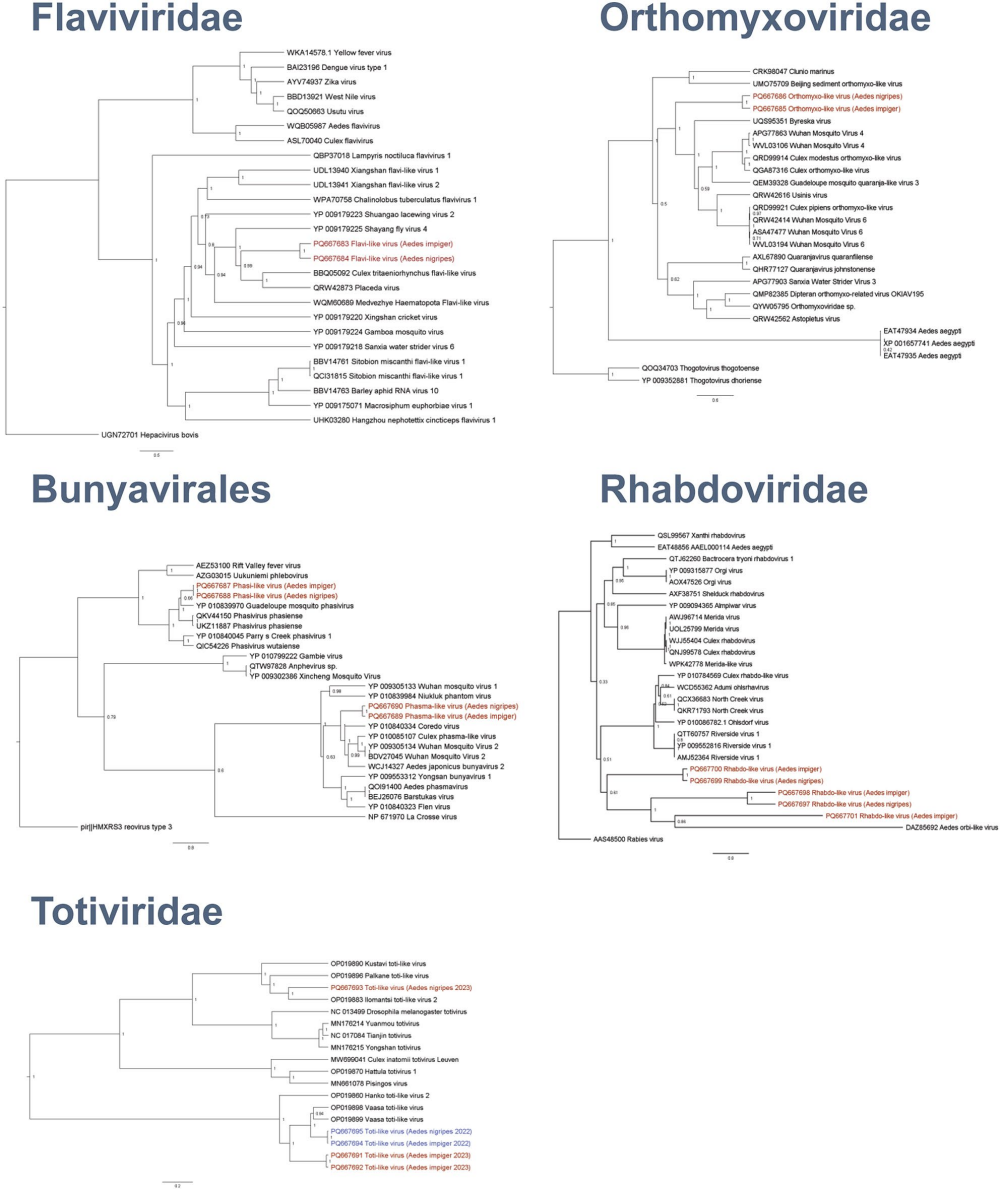
Phylogenetic analysis. Bayesian phylogenetic relationships of virus sequences identified in this study (labelled red and blue) with published sequences. The analysies were based on polyprotein (Flaviviridae, Totiviridae), nucleocapsid (Bunyavirales) or nucleoprotein (Orthomyxoviridae, Rhabdoviridae) amino acid sequences. Node labels represent posterior probabilities and accession numbers are shown.

Sequence alignments (Fig. 3) were produced using MAFFT v7.471 and the resulting alignment was imported into BEAST (v1.10.4). A Bayesian phylogenetic tree was produced using the Blosum62 amino acid substitution model and 10,000,000 Markov chain Monte Carlo generations. Log files were analysed in Tracer v1.7.1 to check the effective sample size and a 10% burn-in was included (TreeAnnotator v.1.10.4) before being visualised and annotated in FigTree v1.4.4.

**Fig. 4:**
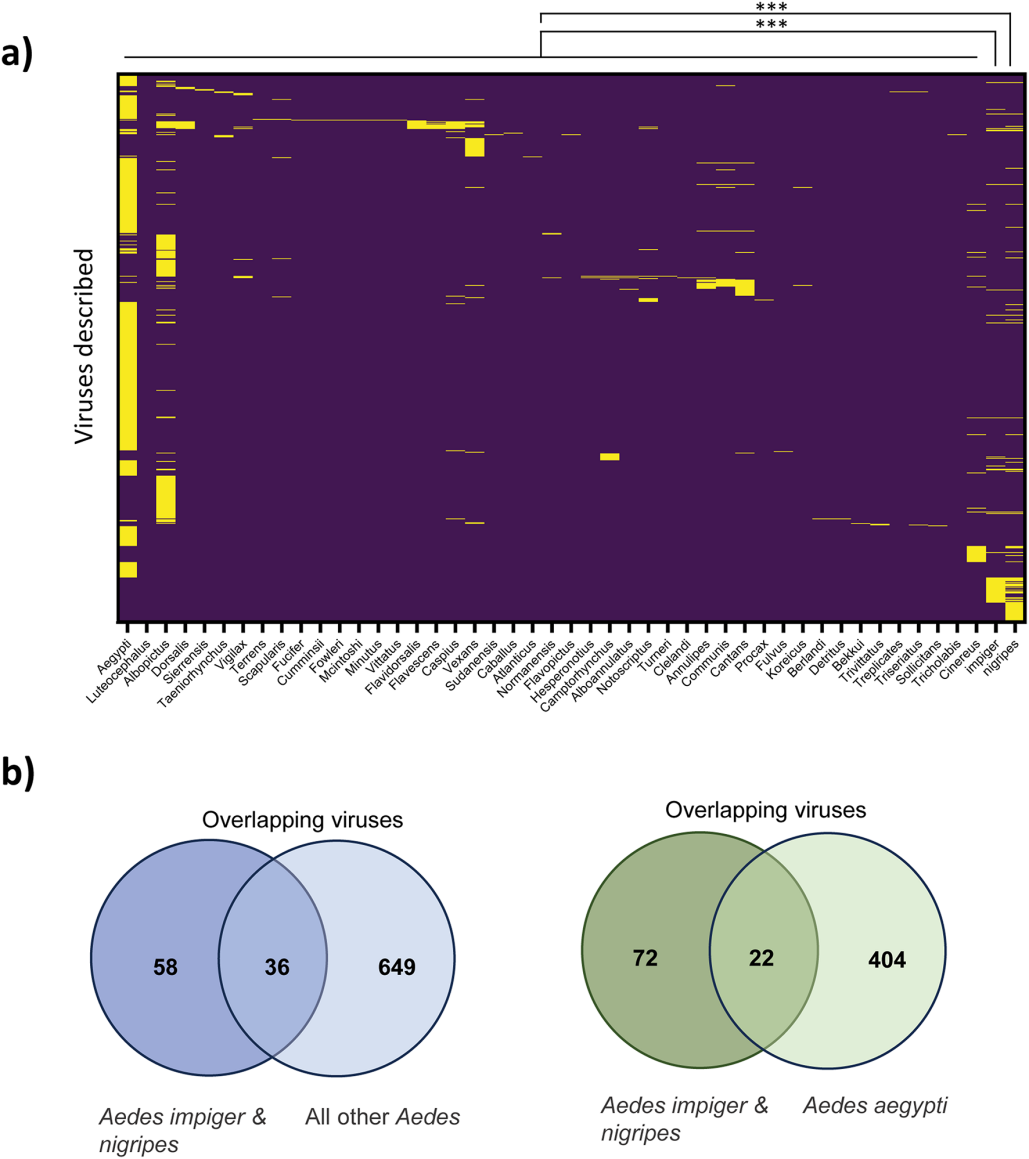
The virome of *Aedes impiger* and *Aedes nigripes* is distinct from other *Aedes* species. **(a)** Heatmap comparing all assigned virus reads in both *Aedes impiger* and *Aedes nigripes* (*n* = 94) with a list of viruses (*n* = 685) published to infect *Aedes spp.* as summarized by Moonen et al. 2023. Two-way Anova with ***P < 0.001 **(b)** Overlap of viruses found between indicated species.

For the heatmap in Fig. 4 a list of viruses for all assigned reads (BLASTX) for both *A. nigripes* and *A. impiger* was added to the database assembled by Moonen et al.^23^. Detections versus non-detections for all viruses and *Aedes* species in the database were calculated with Tidyverse (version 1.3.1) in R (version 4.4.1). A heatmap was created with GraphPad Prism (version 8.4.2).

## Results

### Aedes impiger was the dominant species collected in Kangerlussuaq

Previous surveys in western Greenland suggested that *A. nigripes* was the only mosquito species present^19^. In July 2022 and July 2023, we trapped a total of 75 and 70 specimen, respectively, near Kangerlussuaq (Fig. 1a and b). The mosquitoes trapped in 2022 served as a pilot study to assess the best methods of preservation, transport, species identification, RNA extraction and metagenomic analysis. Species identification by morphology proved to be challenging due to the poor sample conditions after transport and the minor visible differences between the most common species described for Greenland (Fig. 1c). Species identification using the *cox*1 partial sequence, effectively discriminated the species present provided the quality of extracted nucleic acids was high. This identified the majority of mosquitoes collected in 2022 as *Aedes impiger*. Of the 75 Diptera trapped, 4 were excluded (3 midges, 1 fly), 37 were identified as *Aedes impiger* (52%), 5 as *Aedes nigripes* (7%), with the remaining 29 samples unidentifiable, due to poor DNA quality and failure to amplify the *cox*1 amplicon. Consequently, all mosquitoes sampled in 2023, (*n* = 70) were individually homogenised in DNA/RNA Shield immediately after trapping. This improved the quality of the nucleic acid extracted after transport, leading to 93% of samples identifiable by DNA barcoding (Fig. 1c). 49 mosquitoes were identified as *A. impiger* (70%) and 16 as *A. nigripes* (23%). No other mosquito species were identified in either field survey.

### Virus diversity in Arctic mosquitoes

Mosquitoes were pooled based on sampling date and species (Supplementary Tables 1 and 2) and submitted to NGS. The majority of reads from specimens trapped in 2022 mapped to host and bacterial genomes, due to poor nucleic acid quality caused by the challenging storage and transport conditions. For mosquitoes trapped in 2023, the majority of reads mapped to eukaryotes and bacteria (Fig. 2a). However, a proportion of reads mapped to a variety of virus families representing positive and negative single-stranded RNA viruses, double-stranded RNA viruses as well as retroviruses (Fig. 2b). Many of the sequences mapped to viruses that have no assigned order or family, others to assigned virus families but with no ascribed genus. The highest percentage of virus reads for both *A. impiger* and *A. nigripes* mapped to non-genus specific reads in the *Totiviridae*, *Chrysoviridae* and *Flaviviridae* families. Most virus families were detected consistently through the sampling period. However, certain virus families, for example Orthophasmavirus, were only detected in a single mosquito pool. Although the identified virus families significantly overlapped between *A. nigripes* and *A. impiger*, especially in the top 15 virus families detected (including 90% and 99% of total viral reads, depending on sampling date and species), they also encompassed their own individual virome footprint (Fig. 2c).

### Phylogenetic analysis reveals the identification of novel viruses

Sequences mapping to those virus families comprising virus species with known zoonotic potential were analysed phylogenetically (Fig. 3, Supplementary Table 4). For all contigs analysed, we aimed to always phylogenetically represent the homologous sequence obtained from both *Aedes* species. In few cases this led to contigs of sizes < 1000 bp or supported by only few reads being included in the analysis (details see Supplementary Table 4). Within the family of *Flaviviridae*, two novel flavi-like virus sequences were identified, with their polyproteins displaying 35% similarity to the nearest published polyprotein (Fig. 3). This was a flavi-like virus derived from *Culex tritaeniorhynchus* (Protein Accession Number: BBQ05092). Similarly, two novel orthomyxo-like sequences were identified within the family of *Orthomoyxoviridae* (Fig. 3). Their nucleoprotein comprised only 36% identity to that of Byreska virus (Protein Accession Number: UQS95351), the nearest published sequence. Within the order of *Bunyavirales*, we discovered two novel phasiviruses as well as two novel phasmaviruses (Fig. 3). The Phasivirus sequences shared 36% and 66% identity with nucleocapsid sequences belonging to Guadeloupe mosquito phasivirus (Protein Accession Number: YP_010839970) and Coredo virus (Protein Accession Number: YP_010840334), respectively. Within the family *Totiviridae*, we discovered novel nucleocapsid sequences (Fig. 3) with 68% shared amino acid sequence to Vaasa toti-like viruses (Protein Accession Number: OP019898 and OP019899) and RNA-dependent RNA polymerase with 89% shared amino acid sequence. Within the family of Rhabdoviridae, we also discovered three distinct nucleoprotein sequences, two of which were present in both *A. nigripes* and *A. impiger* (Fig. 3).

### Mosquitoes in Greenland host a unique Virome

To emphasize the uniqueness of the virome of *A. impiger* and *A. nigripes*, we compared our findings to a database of viruses published for other *Aedes* species (compiled by Moonen et al.^23^). The heatmap (Fig. 4a) reveals that the majority of sequences derived from Greenland mosquitoes were unique to *A. impiger* and *A. nigripes*, with only 36 (of a total of 94, 38%) assigned viruses overlapping with viruses published for other *Aedes spp*. (Fig. 4b). Only 22 (23%) overlapped with *Aedes aegypti*, the Aedes mosquito with the best characterized virome (Fig. 4b). A Two-way Anova revealed a p-value < 0.0001 for the comparison of both *A. impiger* and *A.nigripes* with all other *Aedes* species, with the exception of the comparison of *A. impiger* with *A. cantans* (*p* = 0.0008). This makes the virome composition of *A. cantans* the most similar published virome composition to that of the mosquitoes we sampled near Kangerlussuaq. This is additionally interesting, as *A. cantans* is geographically distributed across the Palaearctic, suggesting that mosquitoes from similar ecological habitats might share similar viruses.

## Discussion

*Aedes nigripes* is the most abundant and most widely distributed mosquito in the Arctic^24^. Despite this, where a definitive identification could be made, *A. impiger* was the most frequently sampled mosquito at the Kangerlussuaq site. The circumpolar distribution of *A. nigripes* makes it the most widespread and northernmost mosquito species in the Arctic region^25^. However, surprisingly little is known about the viruses harboured by this and other indigenous mosquito species. Our study is the first metagenomic exploration of the viromes of *A. impiger* and *A. nigripes* in Greenland. In our study, the majority of identified virus sequences were found in both *A. impiger* and *A. nigripes.* They also harboured a set of sequence reads assigned to viruses that were unique to each species, implying a distinct virome, although this varied over the sampling period. Due to our limited sample size, future studies are needed to explore the differences and similarities with the virome of other Aedes species in more depth, and to determine how significant the virome difference between *A. impiger* and *A. nigripes* is. Larger datasets, including metagenomic data from other locations in Greenland will be crucial to answer remaining questions. Since insect-specific viruses can affect the replication and transmission efficiency of zoonotic viurses, future studies should also investigate if the virome differences observed cause functional consequences for the vector competence of A. *impiger* and A. *nigripe*s.

A consistent finding in almost all mosquito pools was the presence of sequences with high identity with viruses detected in Finland^16^. These were reported to be derived from *A. excrucians*, also referred to a *Ochlerotatus excrucians*. Totiviridae sequences have also been detected in mosquitoes in extreme northerly locations such as Western Siberia^17^. However, this family of double-stranded RNA viruses has been associated with a range of arthopods including tabanid flies^26^ and *Culicoides* midges^27^. We also found that the *A. impiger* and *A. nigripes* virome comprises of sequences belonging to many of the major virus families described for other mosquito species. However, sequence identity of our assembled contigs with sequences published for other mosquito species was low, sometimes below 35% identity at the on amino acid level. This may explain why the few studies that have previously attempted to characterise the virome of Arctic mosquitoes by PCR often failed to detect viruses^19,20^.

Even less is known about the vector competence of *A. impiger* and *A. nigripes* and the potential impact of novel viruses arriving in Arctic ecosystems or a potential change in global distribution due to a changing climate. *Aedes nigripes* has been suggested to transmit Getah virus in Siberia^28^, and there was evidence of infection with Anadyr virus and Chatanga viruses^19^. *Aedes impiger* is known to feed on humans and can productively produce eggs and oviposit after a human blood meal^29^. These observations indicate a realistic risk that these Arctic mosquitoes are capable of transmitting viruses with zoonotic potential. Our findings further support the capability of *A. nigripes* and *A. impiger* to replicate viruses belonging to a range of different virus families, including (-)ssRNA, (+)ssRNA, dsRNA and retroviruses. However, none of the viruses detected are closely related to known viruses with zoonotic potential and are likely insect-specific. Further studies will need to functionally characterise the viruses we detected, with regard to their replicative capabilities, potential to cause disease and impact on the replication and transmission of other, better characterized arboviruses.

Recent publications suggest that a mosquito virome is less driven by location, but by species^30^. In our study, we describe the virome of two uncharacterized mosquito species in a remote location. Consequently, we cannot know whether the unique virome we observed is driven by the isolated location or the species assemblage, although we note that many virus sequences were present in both species. Mosquitoes currently inhabiting secluded locations, such as the Arctic, might be susceptible to infection and transmit different viruses described in similar species if pathogens or mosquitoes are expanding or changing their distribution^3^. Understanding and being able to predict which viruses will most likely be able to be transmitted by which mosquito species, based on their described virome, would help to estimate the risk of zoonotic disease transmission and inform policies. As our heatmap shows, there are still numerous gaps in our knowledge about the virome of different Aedine mosquitoes, while most studies concentrate on only very few species. Although it is unclear what risk the viruses assigned to the sequences we observed represent to plants, animals, or the human population, our results highlight that Arctic mosquitoes have a distinct virome and could support replication of viruses with zoonotic potential. As a changing climate will increase the likelihood of novel viruses arriving in remote locations and mosquitoes currently restrained to remote locations migrating into different ecosystems, this will most likely impact animal and public health. In light of the predicted expansion of both vectors and their viruses in a changing climate, this should be considered in future risk management plans.

## Electronic supplementary material

Below is the link to the electronic supplementary material.

Supplementary Material 1

## Data Availability

All virus contigs shown in the phylogenetic analysis have been deposited in GenBank under accession numbers: PQ667683 - PQ667701. COX-1 sequences of Aedes impiger and A. nigripes have been deposited in GenBank under accession numbers: PQ645069 - PQ645072. The raw sequence reads generated in this study are available at the NCBI Sequence Read Archive (SRA) database under BioProject PRJNA1230858; BioSamples SAMN47195350 - SAMN47195360.
