# Supplementary material for: Metagenomic analysis of mosquitoes from Kangerlussuaq, Greenland reveals a unique virome
Source: Sci Rep. 2025 May 17;15:17141. doi: 10.1038/s41598-025-01086-z (PMC12085661; doi:10.1038/s41598-025-01086-z)
Supplement: Supplementary file 1 — Supplementary Material 1 [file 41598_2025_1086_MOESM1_ESM.docx]

**Supplementary information**

**Metagenomic analysis of Greenland mosquitoes reveals a unique virome**

**Schilling et al. 2025**

Supplementary Table 1. Mosquito pools 2022 based on species and date of trapping for next generation sequencing.

| Year | Pool ID | Sampling date | Species | n= |
| --- | --- | --- | --- | --- |
| 2022 | 1 | 07.07. | *A. impiger* | 3 |
| 2022 | 2 | 11.07. | *A. impiger* | 3 |
| 2022 | 3 | 16.07. | *A. impiger* | 2 |
| 2022 | 4 | 19.07. | *A. impiger* | 4 |
| 2022 | 5 | 23.07. | *A. impiger* | 3 |
| 2022 | 6 | 23.07. | *A. impiger* | 4 |
| 2022 | 7 | 24.07. | *A. impiger* | 4 |
| 2022 | 8 | 25.07. | *A. impiger* | 3 |
| 2022 | 9 | 25.07. | *A. nigripes* | 2 |
| 2022 | 10 | 23.07. | *A. nigripes* | 1 |
| 2022 | 11 | 07.07. | *A. nigripes* | 2 |
| 2022 | 12 | 05.07. | *A. impiger* | 4 |

Supplementary Table 2. Mosquito pools 2023 based on species and date of trapping for next generation sequencing.

| Year | Pool ID | Sampling date | Species | n= |
| --- | --- | --- | --- | --- |
| 2023 | 1 | 04.07. | *A. impiger* | 8 |
| 2023 | 2 | 08.07. | *A. impiger* | 8 |
| 2023 | 3 | 11.07. | *A. impiger* | 8 |
| 2023 | 4 | 14.07. | *A. impiger* | 7 |
| 2023 | 5 | 16.07. | *A. impiger* | 10 |
| 2023 | 6 | 22.07. | *A. impiger* | 6 |
| 2023 | 7 | 04.07. | *A. nigripes* | 2 |
| 2023 | 8 | 08.07. | *A. nigripes* | 2 |
| 2023 | 9 | 14.07. | *A. nigripes* | 2 |
| 2023 | 10 | 19.07. | *A. nigripes* | 7 |
| 2023 | 11 | 22.07. | *A. nigripes* | 3 |

Supplementary Table 3. Quality of sequencing reads produced for mosquito pools 2023.

| Pool ID | Total Reads | Passed QC | Duplicate Compression Ratio (DCR) | Passed Filters |
| --- | --- | --- | --- | --- |
| 1 | 39,649,018 | 57.73 | 1.57 | 443.724 |
| 2 | 47,539,660 | 58.63% | 1.58 | 490,014 |
| 3 | 67,914,008 | 59.15% | 1.66 | 546,816 |
| 4 | 62,949,466 | 55.38% | 2.13 | 594.412 |
| 5 | 82,997,442 | 61.23% | 2.05 | 998,348 |
| 6 | 51,056,348 | 59.49% | 1.55 | 756,024 |
| 7 | 69,212,232 | 60.18% | 1.53 | 596,888 |
| 8 | 61,005,208 | 61.54% | 2.05 | 345,506 |
| 9 | 67,973,012 | 63.08% | 1.71 | 418,192 |
| 10 | 62,970,778 | 60.73% | 1.18 | 1,267,54 |
| 11 | 96,544,400 | 64.37% | 1.55 | 1,482,96 |

Supplementary Table 4. Breakdown of detections per pool as well as sequencing information on phylogenetically analysed contigs.

| **Virus family or order** | **Detections (nr contigs/#pool)** | **GenBank ID** | **Putative viral gene (partial sequence)** | **Length(nt)** | **Reads** | **Average %ID** |
| --- | --- | --- | --- | --- | --- | --- |
| Bunyavirales | 1/#1  9/#2  1/#3  1/#4  2/#5  2/#6  2/#7  1/#8  2/#9  3/#10  6/#11 | PQ667687 | nucleocapsid | 2102 | 82 | 64.4 |
|  |  | PQ667688 | nucleocapsid | 2697 | 258 | 63.5 |
|  |  | PQ667689 | nucleocapsid | 2286 | 976 | 46.6 |
|  |  | PQ667690 | nucleocapsid | 1010 | 17 | 63.3 |
| Flaviviridae | 5/#1  1/#2  0/#3  0/#4  2/#5  0/#6  12#7  0/#8  1/#9  1/#10  1/#11 | PQ667683 | polyprotein | 7214 | 793 | 34.5 |
|  |  | PQ667684 | polyprotein | 21615 | 7335 | 35 |
| Orthomyxoviridae | 0/#1  0/#2  0/#3  0/#4  0/#5  1/#6  0/#7  0/#8  0/#9  2/#10  3/#11 | PQ667685 | nucleoprotein | 2187 | 87 | 26.3 |
|  |  | PQ667686 | nucleoprotein | 3525 | 362 | 27.9 |
| Rhabdoviridae | 4/#1  6/#2  6/#3  2/#4  6/#5  8/#6  8/#7  0/#8  2/#9  18/#10  12/#11 | PQ667697 | nucleoprotein | 3109 | 123 | 41.5 |
|  |  | PQ667698 | nucleoprotein | 471 | 17 | 60.9 |
|  |  | PQ667699 | nucleoprotein | 3933 | 469 | 38.4 |
|  |  | PQ667700 | nucleoprotein | 344 | 5 | 51.0 |
|  |  | PQ667701 | nucleoprotein | 2682 | 147 | 59.2 |
| Totiviridae | 1/#1  1/#2  2/#3  2/#4  3/#5  3/#6  2/#7  2/#8  1/#9  5/#10  1/#11 | PQ667691 | polyprotein | 3781 | 256 | 23.2 |
|  |  | PQ667692 | polyprotein | 3187 | 172 | 29.6 |
|  |  | PQ667693 | polyprotein | 6360 | 2200 | 39.3 |
|  |  | PQ667694 | polyprotein | 1189 | 133 | 59.0 |
|  |  | PQ667695 | polyprotein | 1354 | 554 | 77.3 |
